# Characteristic Mode Analysis-Based Compact Dual Band-Notched UWB MIMO Antenna Loaded with Neutralization Line

**DOI:** 10.3390/mi13101599

**Published:** 2022-09-26

**Authors:** Praveen Kumar, Tanweer Ali, Manohara Pai MM

**Affiliations:** 1 Department of Electronics and Communication Engineering, Manipal Institute of Technology, Manipal Academy of Higher Education, Manipal 576104, India; 2 Department of Information and Communication Technology, Manipal Institute of Technology, Manipal Academy of Higher Education, Manipal 576104, India

**Keywords:** neutralization line, isolation, MIMO, UWB, band notch, diversity parameters

## Abstract

The advancement of electronic gadgets makes it possible for a device to be multipurpose, which calls for attributes such as compactness and larger bandwidth, with improved data transfer rate. This paper introduces the compact, closely placed two-port dual band-notched UWB antenna using a neutralization line as a decoupling structure. The projected antenna design comprised a circle and rectangle embedded monopole radiator with the defected ground structure to attain the UWB spectrum. Further dual notches are attained by carving the U-shape and inverted U-shape slots on the feedline and radiator. The dual band-notched UWB antennas are placed with the separation of 3.8 mm (0.04 λ; λ is computed using 3.4 GHz frequency). The coupling effect between the close proximity elements is decoupled using the neutralization line. The presented antenna has overall dimensions of 21.5 × 28 × 1.6 mm^3^ (0.24 × 0.31 × 0.01 λ^3^) and exhibits S11 below −10 dB from 3.4–11.9 GHz, with isolation better than 16 dB throughout the impedance bandwidth. The antenna also provides frequency band rejection of 4.5–5.3 GHz and 7.2–9 GHz covering the WLAN and entire X-band satellite communication. The projected antenna is explored through characteristic mode analysis, time-domain characteristics, and MIMO diversity features to analyze the effectiveness and usefulness of the antenna. The group delay is less than 1 ns except for the frequency rejection band and fidelity factor greater than 0.96. The projected antenna exhibits MIMO diversity metrics ECC < 0.3, DG > 9.6 dB, MEG < −3 dB, TARC < −10 dB, CCL < 0.3 bps/Hz, and ME < −2 dB across the operational frequency, except for the notched bands. The designed two-port antenna is validated by printing on an FR4 substrate. The simulated and measured findings are in line with and appropriate for MIMO wireless applications.

## 1. Introduction

The advancement of portable electronic devices is primarily reliant on wireless data transfer technology. These smart electronic devices may perform a variety of functions, such as sensing, remote access, automation, health monitoring, and so on. Wireless communication needs a large bandwidth ensuring uninterrupted data transfer to take advantage of these features. The ultrawideband (UWB) technology addresses the requirement for increased bandwidth. In UWB, an unlicensed frequency band of 3.1 to 10.6 GHz is designated for commercial use [1]. The inclusion of the 7.5 GHz bandwidth in the UWB system has gained attention and led to significant advancements in wireless communication systems. The UWB technology communicates via shorter electromagnetic pulses. The UWB antenna design plays a crucial role in the UWB system as it has to meet the precise time and frequency domain characteristics over the impedance bandwidth. The microstrip antenna is very popular due to its ease of installation in front-end radio frequency design. Monopole antennas, in particular, are highly promising for UWB wireless communication because of their advantages of compactness, high bandwidth, and low cost [2,3]. The first step in realizing the UWB spectrum is to create a standard rectangular or circular monopole antenna. Adjusting a monopole antenna’s radiating and ground plane structures affects the transmission line characteristics and disrupts the uniform current distribution, resulting in impedance matching over the intended frequency of operation. Various antenna configurations are described in the literature to achieve impedance matching over the UWB band. In [4], the authors present an inverted L-shape with a microstrip feedline and a square slot antenna. The geometrical adjustment of the feedline and slot contributes to achieving an impedance bandwidth of 3.5 to 9.5 GHz. The combination of different geometrical cylinders with coplanar waveguide (CPW) staircase slot ground structure UWB monopole antenna is demonstrated in [5]. The rectangular radiator with arc shape truncation at the lower corner and CPW arrangement used to realize the frequency from 3.1–11.8 GHz is presented in [6]. For the UWB spectrum, an arc-shaped radiator with a lower ground plane composed of parasitic components is illustrated in [7,8]. The extended UWB band monopole antenna consisting of a spline-enhanced based radiator and defected ground structure (DGS) is presented in [9].

Although the UWB system has numerous benefits, including wide bandwidth, it interferes with the existing narrow bands. Conventional antenna design techniques for band rejection characteristics are generally centered on employing a half- or quarter-wavelength filter arrangement. Several other techniques for eliminating single or multiple frequency bands have been reported in the literature, including different structured parasitic elements loading on the radiator and ground plane, metamaterial loading, embedding the filter and antenna, and combining two or more notching techniques as a hybrid approach [10]. The Sprocket gear wheel-shaped radiating patch defected by the arc and rectangular slots to accomplish two frequency band notches is presented in [11]. The fractal UWB antenna in [12] comprises an inverted S and a pair of L-shaped slots and stubs on the radiator to accomplish WLAN and X-band frequency notching. In [13], the WiMAX and WLAN frequency bands are filtered utilizing S and inverted U structure slots on the hexagonal patch and ground plane. The authors in [14] illustrate the WLAN and X-band notching characteristics of the circular ring UWB antenna. Band notching is accomplished using parasitic elements on the radiator and a capacitively-loaded loop on the ground plane. The design and positioning of the notching element on the radiator or ground plane primarily influence the desired frequency of notching.

The need for good reproducibility, i.e., wireless links susceptible to multipath fading and higher data rate, is rising proportionally to the progress of wireless communication technology. The portable gadgets are outfitted with diverse approaches to meet the requirement without extending either the spectrum or the transmission power. The compactness features of devices confine the placement and floor planning of the sub-components. Furthermore, putting several antennas in a small area will cause strong mutual coupling between the antennas. In the literature, a large number of band-notched UWB MIMO antennas have already been documented. In such antenna systems, isolation is enhanced by utilizing different approaches, such as defected ground structure (DGS), decoupling networks, neutralization line (NL), metamaterial loading onto the ground and radiator, incorporating filters, and a combination of two or more decoupling structures as hybrid techniques, as reported in [15,16]. In [17], a two-port UWB fractal antenna with the dimensions of 26 × 35 mm^2^ operating from 2–10.6 GHz was demonstrated. The presented antenna has dual band notch features at 3.5 GHz and 5.2 GHz by embedding a slot and stub on the radiator. The coupling of the antenna is reduced by introducing the strip on the ground plane. An orthogonally arranged two-port circular monopole UWB antenna with dimensions of 25 × 39 mm^2^ is illustrated in [18]. The presented antenna has dual notches at 5.4 GHz and 7.5 GHz by engraving L-shaped slots onto the radiator. The U-shaped branches entrenched in the ground plane reduce the mutual coupling. The electromagnetic band gap (EBG) structure that separates the two CPW-fed UWB antennas to enhance the isolation (>20 dB) is presented in [19]. The presented design has dimensions of 35 × 32 mm^2^ with the S11 curve ranging from 3.4 GHz to 10.4 GHz.

According to the literature, most MIMO antennas use DGS to improve isolation, and the interelement separation is rather large. This work describes a unique two-port UWB MIMO antenna with a decoupling mechanism as a neutralization line. Impedance matching for the UWB spectrum is performed using a hybrid structure composed of a circular monopole with a rectangle as a radiator and a decreased ground plane. The lowered ground plane creates a local current channel, which aids in achieving a broader impedance bandwidth. U-shape slots are carved on the radiator to accomplish dual band-notching at WLAN and X-band frequencies. The band-notched monopole UWB antenna is horizontally recreated with a much less than quarter wavelength separation (i.e., 3.8 mm or 0.04 λ, λ is computed using a lower frequency of 3.4 GHz). The coupling effect among the elements is reduced using a neutralization line. The projected antenna has physical dimensions of 21.5 × 28 × 1.6 mm^3^ (0.24 × 0.31 × 0.01 λ^3^) with an impedance bandwidth of 112% (3.4 to 12.2 GHz) and isolation greater than 16 dB throughout the operating frequencies. Furthermore, the projected antenna is investigated for characteristic mode analysis (CMA), MIMO diversity characteristics, and time-domain features. For experimental validation, the proposed design is printed on an FR4 substrate ($\varepsilon_{r}=4.4,\;\tan\delta =0.02,\;h=1.6\;\mathrm{mm}$).

### Novelty and Contributions

The following are the significant contributions of this work:

1. The efficacious and simple UWB monopole design achieves a reflection coefficient curve of less than −17 dB throughout the entire working frequency range.
2. The controllable dual notch frequencies are achieved at the WLAN and complete X-band (uplink and downlink) satellite frequency bands.
3. To construct the two-port dual-notch UWB antenna, the intended dual-notched UWB antenna is reproduced with a minimum 3.8 mm separation parallelly.
4. The proposed MIMO antenna is evaluated for the characteristic mode analysis (CMA), MIMO diversity features, and time-domain characterization.

The remainder of the study is structured as follows: The detailed antenna design and its evolution are presented in Section 2. Section 3 outlines the decoupling mechanism designed to achieve the optimal isolation of the projected antenna. Section 4 provides the findings of the CMA. The performance characteristics of the projected design are illustrated in Section 5. Section 6 provides concluding remarks.

## 2. Antenna Design

### 2.1. Dual Band-Notched UWB Antenna

**Figure 1:**
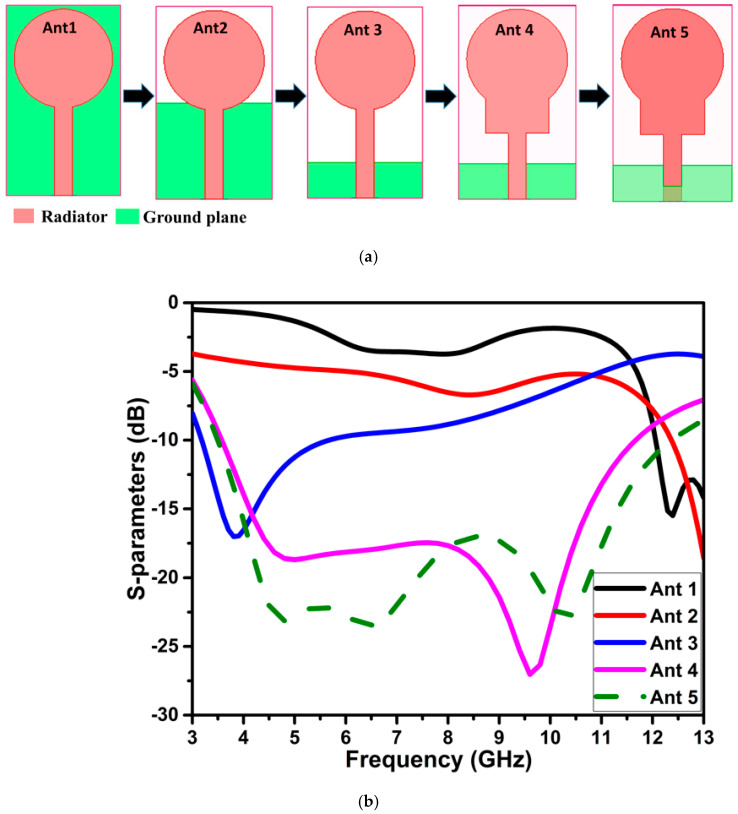
Evolution of UWB antenna: (**a**) structure (**b**) simulated S-parameters curve.

**Figure 2:**
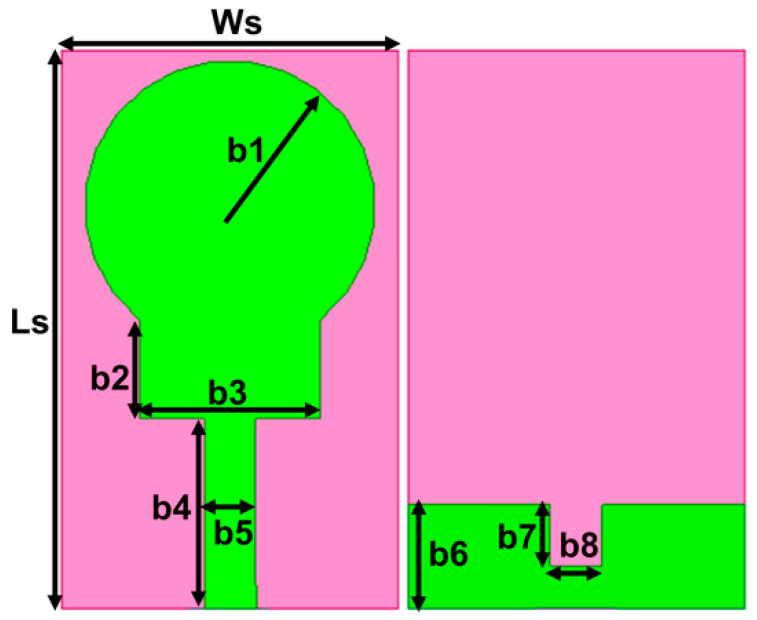
The geometrical information of the projected UWB antenna.

The conventional circular monopole is modified to attain operational frequency in the UWB range. The UWB antenna structure evolution is illustrated in Figure 1a. The simulated S-parameters curve at every stage (ant 1, ant 2, …, ant 5) is depicted in Figure 1b. In the first step, the radius of the circular patch is calculated using the Equations (1)–(3) with a microstrip feedline [20]. This configuration shows the S11 curve below −10 dB from 12 GHz onwards, as shown in Figure 1b for ant 1. To accomplish wider impedance bandwidth, the ground plane of the circular monopole antenna is lowered in response to the impedance bandwidth. The alteration of the ground plane disturbs the uniform current distribution affecting the transmission line properties and quality factor, which helps to attain the broader bandwidth, as shown in Figure 1 for ant 2, and 3, and ant 5. Following the reduced ground plane, the radiator is modified by integrating a rectangular shape between the circle and feedline, as shown in Figure 1 for ant 4. This arrangement provides simulated impedance bandwidth below −20 dB from 3.5 to 12.4 GHz, except for the middle frequencies, where it is below −17 dB. The ant 5 in Figure 1 provides the required UWB spectrum having overall physical dimensions of 21.5 × 13 × 1.6 mm^3^, and uses FR4 as a substrate. The dimensions of the antenna element, stub, slot, ground plane, and feedline of the UWB antenna (ant 5) are represented in Figure 2, and their values are listed in Table 1.

The radius (*r*) of the circular patch is determined by

(1) $$r=\frac{F}{\sqrt{1+\frac{2h}{\pi \varepsilon_{r}F}\;\left[\ln\left(\frac{\pi F}{2h}\right)+1.7726\right]}}\;$$

where *F* is given by

(2) $$F=\frac{8.791\times 10^{9}}{f_{r}\sqrt{\varepsilon_{r}}}\;$$

The effective radius results from the fringing field spreading from the patch border to the ground plane. The fringing field around the circular patch may increase the radius of a circle. Therefore, the effective radius ($r_{eff}$) is determined as shown below

(3) $$r_{eff}=\frac{1.8412\;c}{2\pi f_{r}\sqrt{\varepsilon_{r}}}\;$$

where *c* = 3 × 10^11^ mm/s, $f_{r}$ is the resonating frequency, and $\varepsilon_{r}$ is the dielectric constant of the FR4 substrate.

In the first stage, a circular monopole antenna is designed using Equations (1)–(3) with the specification of $\varepsilon_{r}\;$= 4.4, h = 0.16 cm, and $f_{r}$ = 7.5 GHz.

$$F=\frac{8.791\;\times \;10^{9}}{7.5\;\times \;10^{9}\;\times \;\sqrt{4.4}}=0.6$$

$$r=\frac{F}{\sqrt{1+\frac{2h}{\pi \varepsilon_{r}F}\;\left[\ln\left(\frac{\pi F}{2h}\right)+1.7726\right]}}=\frac{0.6}{\sqrt{1+\frac{2\;\times \;0.16}{\pi \;\times \;4.4\;\times \;0.6}\;\left[\ln\left(\frac{\pi \;\times \;0.6}{2\;\times \;0.16}\right)+1.7726\right]}}$$

r=5.6 mm

$$r_{eff}=\frac{1.8412\;c}{2\pi f_{r}\sqrt{\varepsilon_{r}}}=\frac{1.8412\;\times \;3\;\times \;10^{11}}{2\;\times \;\pi \times 7.5\;\times \;10^{9}\times \sqrt{4.4}}\;5.6\;\mathrm{mm}$$

The radius and effective radius are 5.6 mm. Therefore, a circle with a 5.6 mm radius is chosen with the 50 Ω matched feedline for ant 1 in Figure 1a.

**Figure 3:**
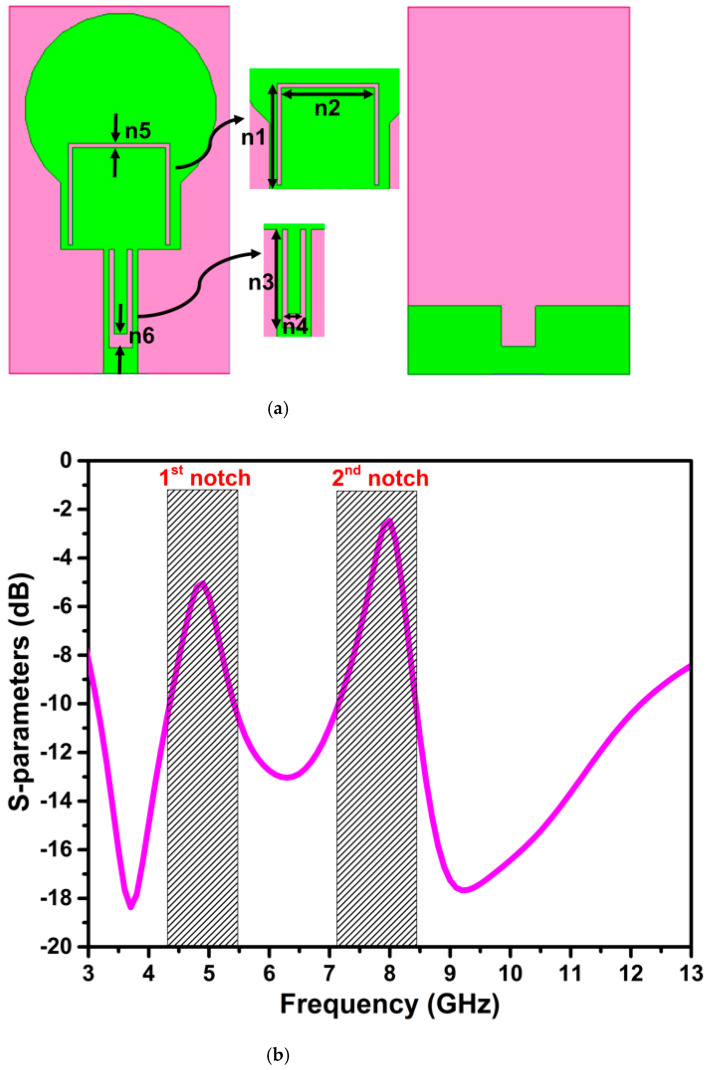
The projected dual band-notched UWB antenna: (**a**) structure (**b**) S-parameter curve.

The UWB spectrum covers the current X-band, WLAN, WiMAX, and other small frequency bands. To prevent interference with the UWB spectrum, narrow-band frequency notching is necessary. In the designed UWB antenna, dual-frequency band-notching is attained using U-shape and inverted U-shape slots on the radiator. The first notching covers the WLAN band from 4.3 to 5.4 GHz, and the second frequency notching from 7.3 to 8.5 GHz covers both the uplink and downlink of X-band satellite communication, as represented in Figure 3a,b. The band notching is performed without affecting the impedance bandwidth other than the intended notch frequencies. The length of the slot is computed using Equations (4) and (5) [10], and their optimal values are listed in Table 2.

(4) $$\;L_{parasitic\;element}=\frac{\lambda_{g}}{2}\;$$

where $\lambda_{g}$ is the guided wavelength and is equal to $\lambda_{g}=\frac{\lambda_{0}}{\sqrt{\varepsilon_{eff}}}$

(5) $$∴L_{parasitic\;element}=\frac{\lambda_{0}}{2\sqrt{\varepsilon_{eff}}}=\frac{c}{2f_{r}\sqrt{\varepsilon_{eff}}}\;$$

where $\varepsilon_{eff}$ is the effective dielectric constant, $f_{r}$ is the resonant frequency in GHz, and *c* is the speed of light.

$$L_{inverted\;U-shape\;slot}=2\left(n1\right)+n2=2\left(6\right)+6=18\;\mathrm{mm}$$

$$f_{r\;U-shpe\;slot}=\frac{c}{2L_{inverted\;U-shape\;slot}\sqrt{\varepsilon_{eff}}}=5\;\mathrm{GHz}$$

$$L_{U-shape\;slot}=2\left(n3\right)+n4=2\left(5\right)+1.4=11.4\;\mathrm{mm}$$

$$f_{r\;U-shpe\;slot}=\frac{c}{2L_{U-shape\;slot}\sqrt{\varepsilon_{eff}}}=8\;\mathrm{GHz}$$

**Figure 4:**
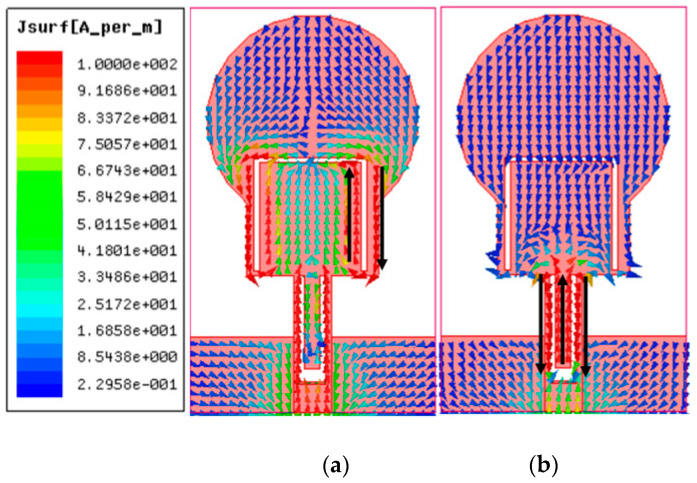
The current distribution of band-notch UWB antenna at (**a**) 5 GHz, (**b**) 8 GHz.

**Figure 5:**
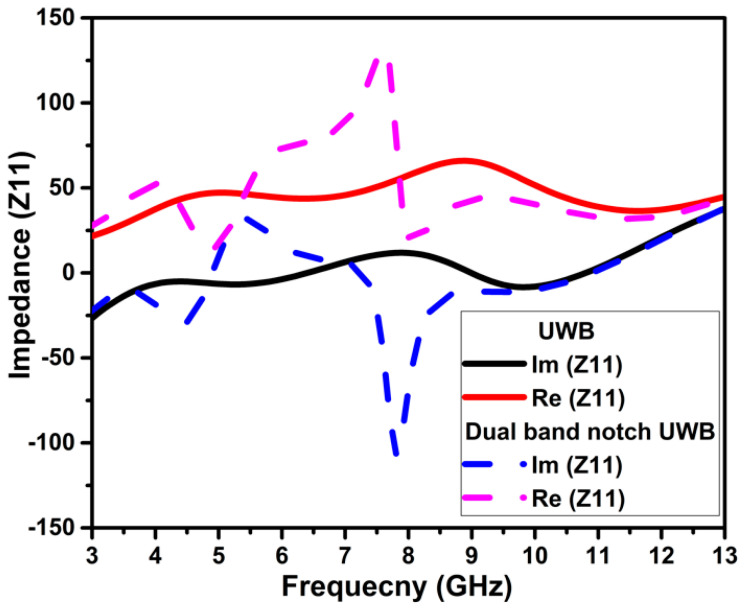
Impedance curve of the UWB and UWB notch antenna.

The center frequency and bandwidth of the notching frequency are controlled by altering the physical parameters of the slots. At the notch frequencies, the U-shape slots on the radiator attract the maximum current and neutralize the current by creating the same magnitude and out-of-phase currents within the slots, as illustrated in Figure 4. Therefore, nothing radiates from the radiator to the free space. Further, the effectiveness of the slots introduced for notching is witnessed by the impedance mismatching at the notched bands in the impedance curve (Z11), as in Figure 5, and the negative gain of −5.62 dB and −5.72 dB at the center frequency of the notched bands 5 GHz and 8 GHz, respectively.

### 2.2. Parametric Analysis

A parametric analysis is used to investigate the variation in dimensions of the projected antenna as well as its influence on antenna properties. This section displays a thorough antenna construction study in order to achieve UWB and dual band-notch frequency characteristics. Initially, a circular monopole antenna is designed with the full ground plane. To attain a wider impedance bandwidth, the length of the ground plane (b6) is reduced successively with a step size of 4 mm. The reduction in a ground plane influences the Q-factor due to the alteration of transmission line properties and increases the operating bandwidth.

**Figure 6:**
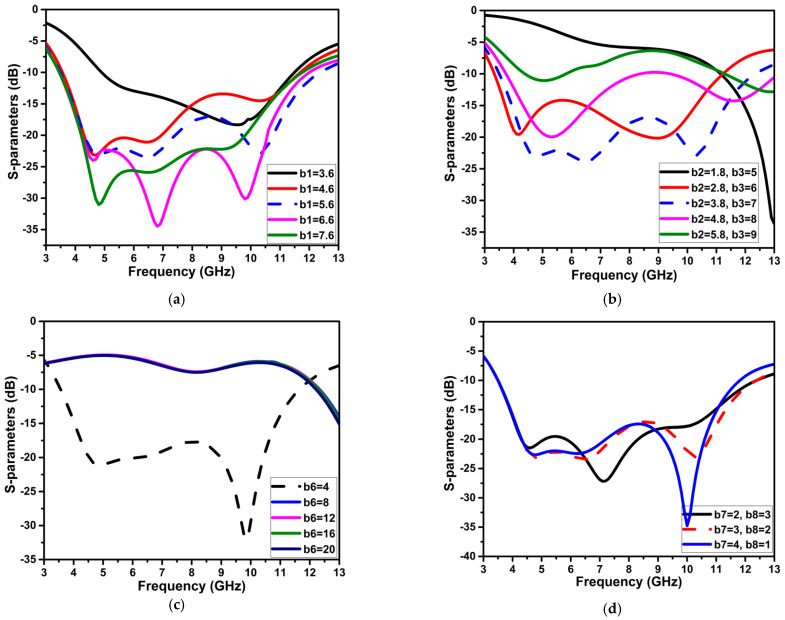
The variations in the parameters of the UWB antenna and their impact on the S11 curve (**a**) b1, (**b**) b2, and b3 (**c**) b6, (**d**) b7, and b8.

Further, to obtain the UWB spectrum, the patch’s radius (b1) varies from 3.6 mm to 7.6 mm. The optimal radius is chosen as 5.6 mm, considering the compactness of the antenna. A rectangular stub with the dimensions of b2 and b3 is attached to the circle of the patch to enhance impedance bandwidth and reflection coefficient values. Finally, a rectangle slot of b7 and b8 dimensions were carved on the ground plane to improve the S11 curve on the higher frequency side. Figure 6 depicts the changes in various UWB antenna parameters and their influence on the S11 curve. Table 1 shows the ideal values for these parameters.

**Figure 7:**
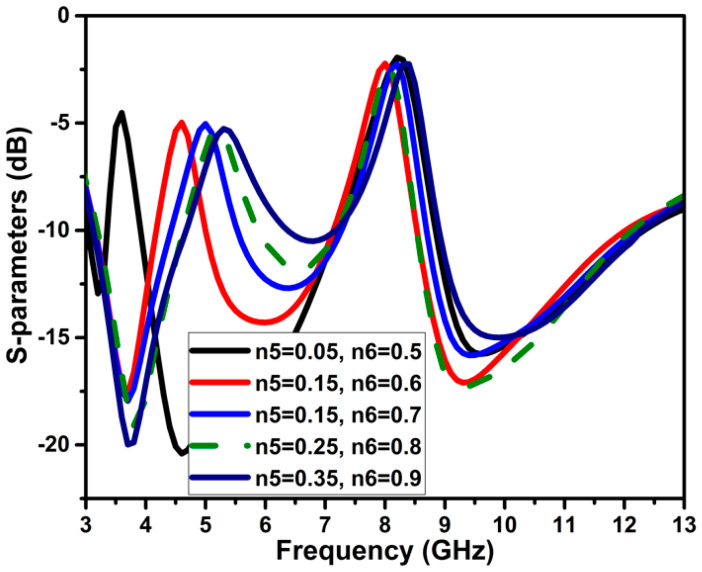
Significance of the variations of n5 and n6 on band notching.

Variations in the n5 and n6 parameters considerably affect frequency band notching, as seen in Figure 7. The n5 is modified in 0.1 mm increments from 0.05 to 0.35 mm, and when the width increases, the notching frequency band moves to the higher frequency. The optimal value of n5 for the required band notch of the WLAN frequency spectrum is 0.25 mm. For the second band notch, n6 is gradually increased from 0.5 to 0.9 mm. To cover the whole X-band satellite communication band notching, the optimal value of n6 is chosen as 0.8 mm; with other values of n6, the bandwidth of notching is expanded.

### 2.3. Two-Port Dual Band-Notched Antenna

**Figure 8:**
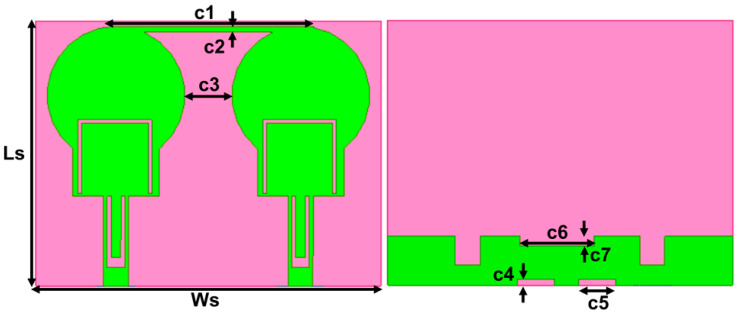
Projected Two-port dual band-notch UWB antenna.

**Figure 9:**
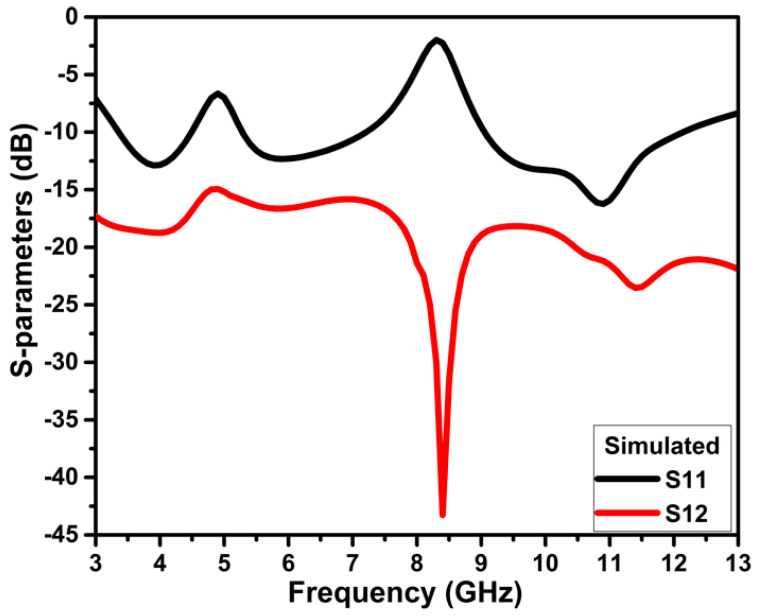
Simulated S-parameters of the projected band-notch UWB MIMO antenna.

The designed dual-notch UWB antenna is replicated horizontally with the minimum edge-to-edge separation of 3 mm to create two port antennas with overall dimensions of 21.5 × 28 × 1.6 mm^3^ (0.24 × 0.31 × 0.01 λ^3^), as represented in Figure 8. The closely placed monopole antennas lead to strong mutual coupling. The coupling between the elements is decoupled using the neutralization line that connects the two symmetrical antennas and DGS. The geometrical information of the projected design is represented in Table 3. The neutralization line is comprised of the horizontal stub that provides an out-of-phase current with identical magnitude to the coupling current resulting in current cancellation and aiding in improving isolation across the impedance bandwidth. Incorporating the neutralization line on the radiator and ground plane increases capacitance, raising the Q-factor and, therefore, resulting in a slight variation in impedance bandwidth between the single element and MIMO antennas. The slots of the same width as C6 and C4 are used to improve the isolation at the lower frequency of 4 GHz. These slots moderately influence the second notch band. The projected two-port antenna operates from 3.4–12.1 GHz with isolation greater than 16 dB, and band notching of 4.5–5.2 GHz and 7.3–8.9 GHz, as illustrated in Figure 9.

## 3. Decoupling Mechanism

**Figure 10:**
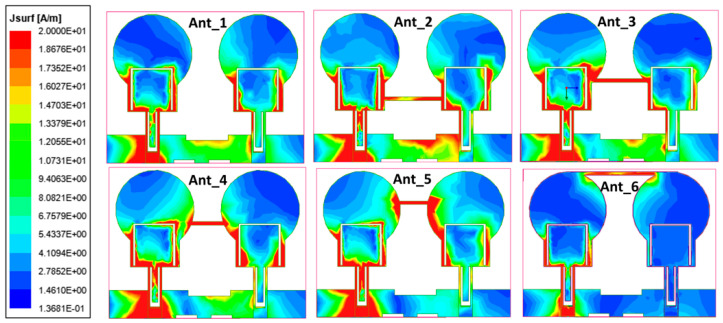
The projected antenna’s current distribution plots of various configurations.

**Figure 11:**
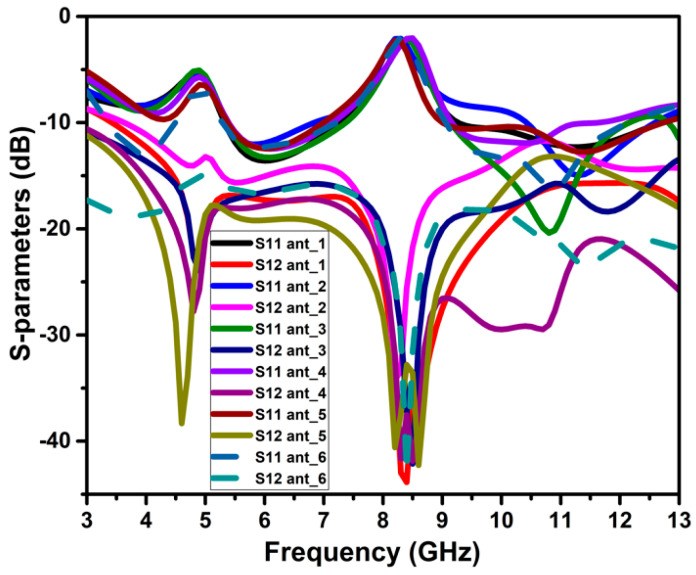
S-parameter curves of the NL evolution.

**Figure 12:**
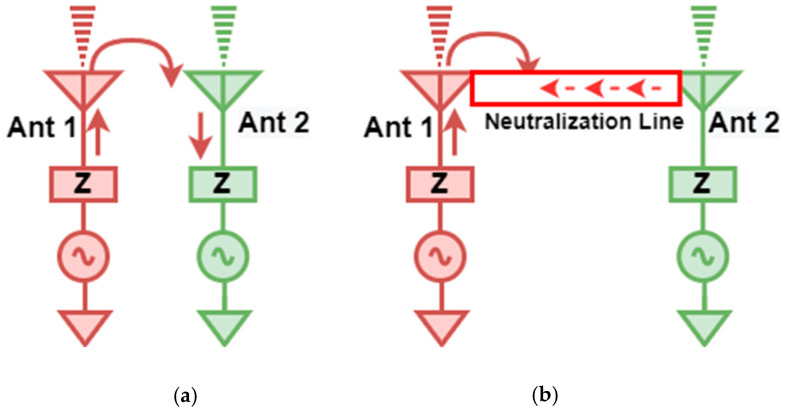
Demonstration of the operation of NL as a decoupling element (**a**) no decoupling (**b**) NL as decoupling structure.

The isolation enhancement process is analyzed by plotting the surface current distribution of the projected antenna, as portrayed in Figure 10. Current distribution graphs are obtained by stimulating one port and terminating the other port with matched conditions. The projected antenna without a neutralization line has a significant coupling effect on the neighboring element, as depicted in Figure 10 ant_1. Positioning the neutralization line (NL) onto the radiator is the most challenging step in using the neutralization line as a decoupling element. NL is originally placed near the middle of the antenna where the largest current concentration is present, based on the current distribution in Figure 10 ant_1. As in Figure 10 ant_2-ant_6, the position of NL is shifted upwards by observing the current distribution plots and their S-parameter curve at a specific placement referred to as ant_1 to ant_6. The S-parameter curve for each arrangement is illustrated in Figure 11. The ant_6 arrangement provides the out-of-phase current to that of the coupling current, resulting in improved isolation, as demonstrated in Figure 12.

It is clear from Figure 11 that ant_1 to ant_5 have poor isolation and reflection coefficient values, particularly at lower frequencies. The ant_6 arrangement provides isolation greater than 16 dB throughout the impedance bandwidth from 3.4–12.1 GHz.

## 4. CMA

Robert J. Garbacz’s 1965 study, “Introduction of modal expansion in electromagnetic scattering on resonance region”, was the first to examine the fundamental theory of CMA [21,22]. The total current that flows through a conducting or radiating structure generated by an incoming electromagnetic field may be described as the weighted sum of N transverse eigencurrents ($J_{i}$) that are geometrical and material reliant while being self-reliant on the excitation vector, according to a key concept of CMA theory [23,24]. The total current can be expressed as in Equation (6)

(6) $$J=\overset{N}{\underset{i=n}{\sum }}\beta_{i}{\;J}_{i\;}\;$$

where $\beta_{i}$ is modal weight coefficients that calculate the impact of each eigencurrent on the overall current. Because each eigencurrent generates its electric field, the overall current generates the total radiated electric field. The modal significance (*MS*) is a significant metric of the CMA that determines each mode’s maximum normalized current strength and influences the radiation properties of that particular mode [25]. The MS can be calculated using Equation (7)

(7) $$MS=\left|\frac{1}{1+j\lambda_{i}}\right|\;$$

where $\lambda_{i}$ is the eigenvalue of the *i*th mode and is related to the eigencurrents using a method of moment matrix ([Z]), as follows:

(8) [Z]=[R]+j[X] 

(9) $$\left[X\right]\;J_{i\;}=\lambda_{i\;}\left[R\right]\;\;J_{i\;}\;$$

where *R* and *X* depict the real and imaginary part of the impedance *Z*. The eigenvalues help analyze the capacitance and inductance values; their significance is listed in Table 4. The phase difference in the electric field and surface current of the antenna is described by the characteristic angle (CA). The CA ($\alpha_{i}$) can be computed using Equation (10)

(10) $$\alpha_{i\;}=180^{{}^{\circ}}-tan^{-1}\lambda_{i\;}\;$$

**Figure 13:**
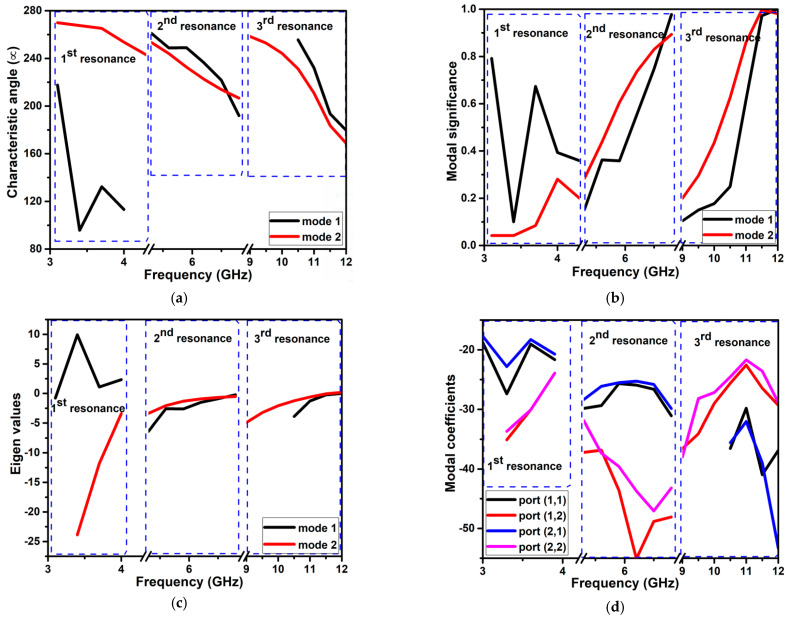
CMA of the projected two-port dual band-notch UWB antenna (**a**) CA (**b**) MS (**c**) eigenvalues (**d**) modal coefficient.

**Figure 14:**
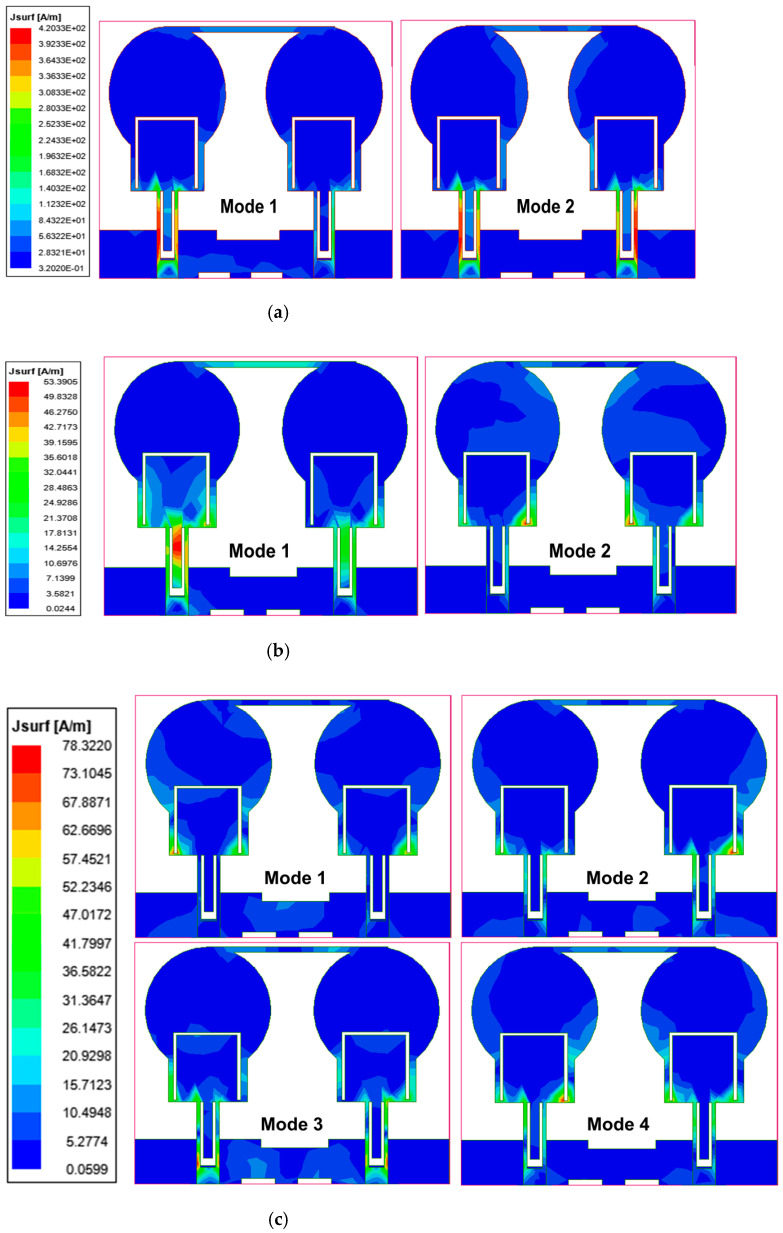
The current distribution of CMA at different modes and frequencies (**a**) 3.7 GHz (**b**) 6.8 GHz (**c**) 11.5 GHz.

CMA was carried out on the projected two-port antenna. At the principal frequency resonance of 3.7 GHz, mode 1 has an MS of 0.7, eigenvalue of 1.09, and CA of 132 degrees. Other modes have lower significance values at this frequency, i.e., MS < 0.1. The nature of this induced mode is inductive. Similarly, at the second resonance frequency of 6.8 GHz, there are two significant modes, 1 and 2. Both the modes are capacitive in nature. Modes 1, 2, 3, and 4 are induced at the subsequent resonance frequency of 11.5 GHz. The first three modes are capacitive, and mode 4 is inductive. The CA, MS, eigenvalue, and modal coefficient curves of the projected design at the working band are illustrated in Figure 13, and their values at the frequencies of 3.7 GHz, 6.8 GHz, and 11.5 GHz are listed in Table 5. Figure 13d shows the modal coefficients between the ports. These coefficients influence the eigencurrent, which generates the overall current and the radiated electric field. The surface current distribution over these frequencies in different modes is illustrated in Figure 14.

## 5. Results and Discussion

**Figure 15:**
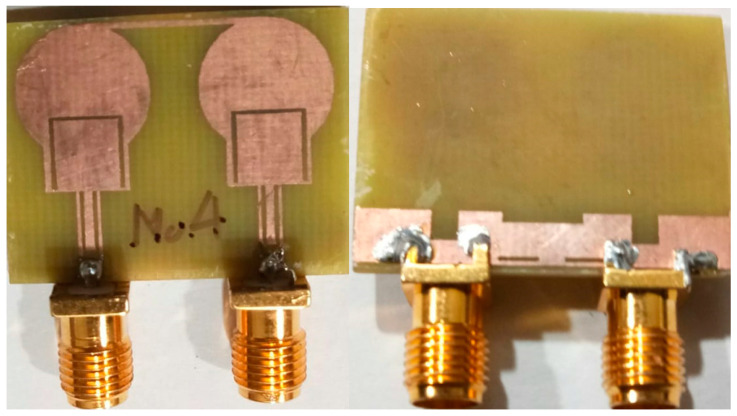
Photos of the fabricated antenna.

The projected two-port dual band-notch UWB antenna is designed using HFSS. To validate the design, the antenna is printed on an FR4 substrate as represented in Figure 15. The simulated and prototype of the designed antenna is examined for scattering parameters, current distribution, radiation characteristics, time-domain features, and MIMO diversity metrics.

### 5.1. Scattering Parameters

**Figure 16:**
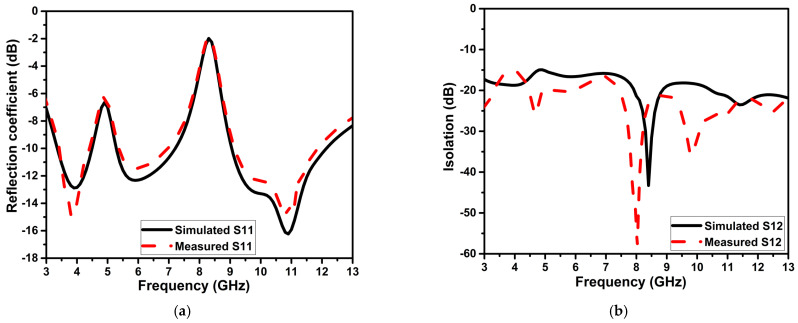
The simulated and experimental S-parameters of the projected antenna (**a**) S11, (**b**) S12.

The S-parameters of the projected antenna design are measured using an Agilent N5247A PNA-X vector network analyzer. The numerical and experimental findings of the S-parameters are depicted in Figure 16. The measured reflection coefficient is below −10 dB from 3.4 to 11.9 GHz, except for the notched frequency bands 4.5–5.3 GHz, 7.2–9 GHz WLAN, and X-band. Throughout the operating frequency of the projected antenna, isolation is better than the 16 dB.

### 5.2. Current Distribution

**Figure 17:**
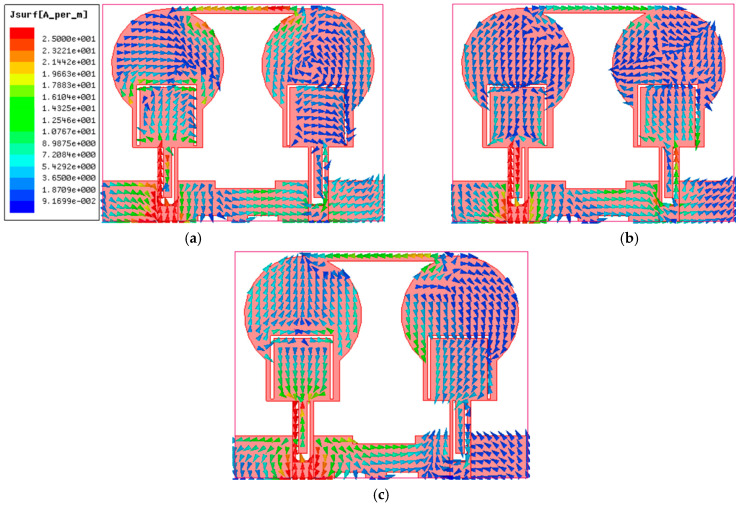
Current distribution graphs of the projected design at (**a**) 4 GHz, (**b**) 6 GHz, (**c**) 10.9 GHz.

The surface current distribution plots for the projected design at 4 GHz, 6 GHz, and 10.9 GHz are as illustrated in Figure 17. These plots are obtained by exciting one port and terminating the other port with the matched conditions. The maximum current concentration is across the feedline, ground plane, and decoupling structure. It can be seen from the figures that the exciting element has a very negligible influence on the neighboring element because NL produces the reverse current to the coupling current, resulting in improved isolation.

### 5.3. Radiation Characteristics

**Figure 18:**
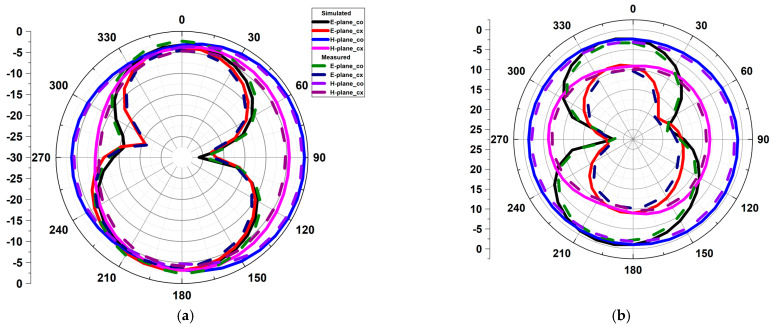

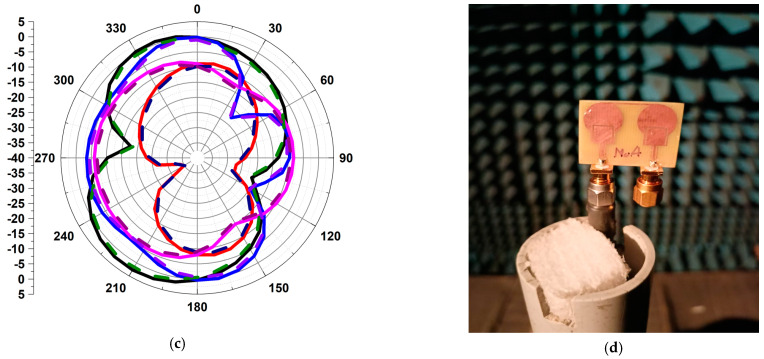
The 2D radiation pattern of the projected design (**a**) 4 GHz, (**b**) 6 GHz, (**c**) 10.9 GHz, (**d**) photo of the measurement.

**Figure 19:**
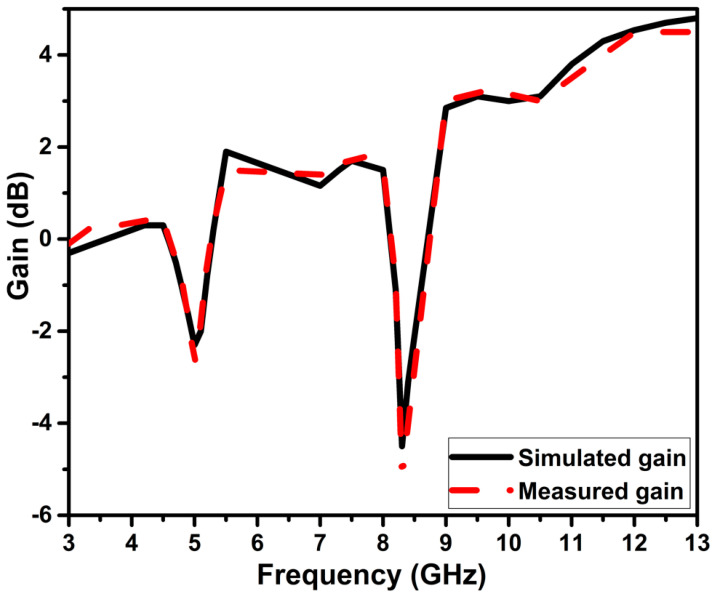
The simulated and measured gain of the projected antenna.

Figure 18 depicts the two-dimensional radiation pattern at the XZ and YZ planes at 4 GHz, 6 GHz, and 10.9 GHz frequencies. These plots are generated by stimulating one port and closing the other with the 50 Ω load, as shown in Figure 18d. In the E-plane and H-plane, the radiation pattern exhibits bidirectional and omnidirectional characteristics, respectively. Figure 19 depicts the measured and simulated gain curve. Band gain is negative at the notch frequency, confirming the operation of the slots used for band notching. At the notch frequency spectrum, the gain is negative and confirms the working of slots used for band notching.

### 5.4. Time-Domain Characteristics

**Figure 20:**
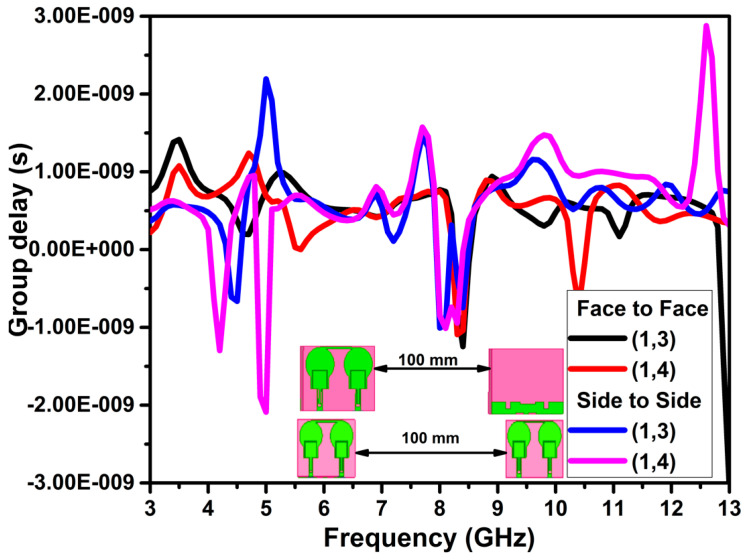
The group delay of the projected antenna in different configurations.

**Figure 21:**
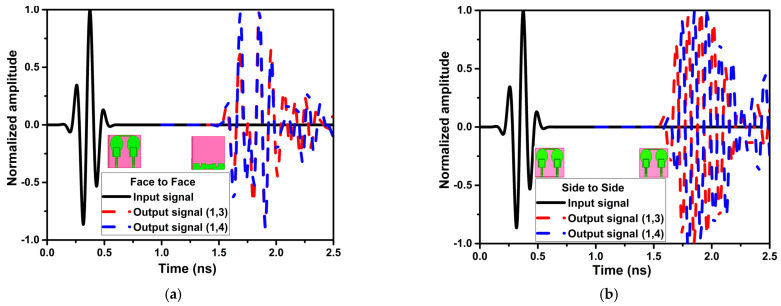
The normalized amplitude curve of the input and output signal (**a**) face to face (**b**) side to side.

The UWB technology transmits and receives data using short pulses. The transmission channel and distance may be the cause of signal distortion and dispersion. As a result, compared to normal antennas, the UWB antenna requires additional parameter evaluation. The two identical antennas are positioned 100 mm apart in face-to-face and side-to-side configurations for the time-domain investigation. Significant time-domain characteristics that govern the phase linearity and form of the received pulses are the group delay (GD) and fidelity factor (FF). Equations (11)–(14) are used to determine the GD and FF [26]. Figure 20 and Figure 21 show the intended design’s GD, normalized input, and output signal. The configurations for the FF, which is for several ports, are shown in Table 6. Except for the notch bands, the projected antenna demonstrates a GD of less than 1 ns and an FF of more than 0.96 in all configurations.

(11) $$GD\left(\tau_{g}\left(\omega \right)\right)=-\frac{d\varphi \left(\omega \right)}{d\omega }\;$$

The phase difference between the ports is characterized by *φ*.

(12) $$\;T_{s}^{n}=\frac{T_{s}\left(t\right)}{\sqrt{\int_{-\infty }^{\infty }{\left|T_{s}\left(t\right)\right|}^{2}dt}}\;$$

(13) $$\;R_{s}^{n}=\frac{R_{s}\left(t\right)}{\sqrt{\int_{-\infty }^{\infty }{\left|R_{s}\left(t\right)\right|}^{2}dt}}\;$$

(14) $$FF=\max\overset{\infty }{\underset{-\infty }{\int }}T_{s}^{n}\left(t\right)\;R_{s}^{n}\left(t+\tau \right)dt\;$$

### 5.5. MIMO Diversity Characteristics

**Figure 22:**
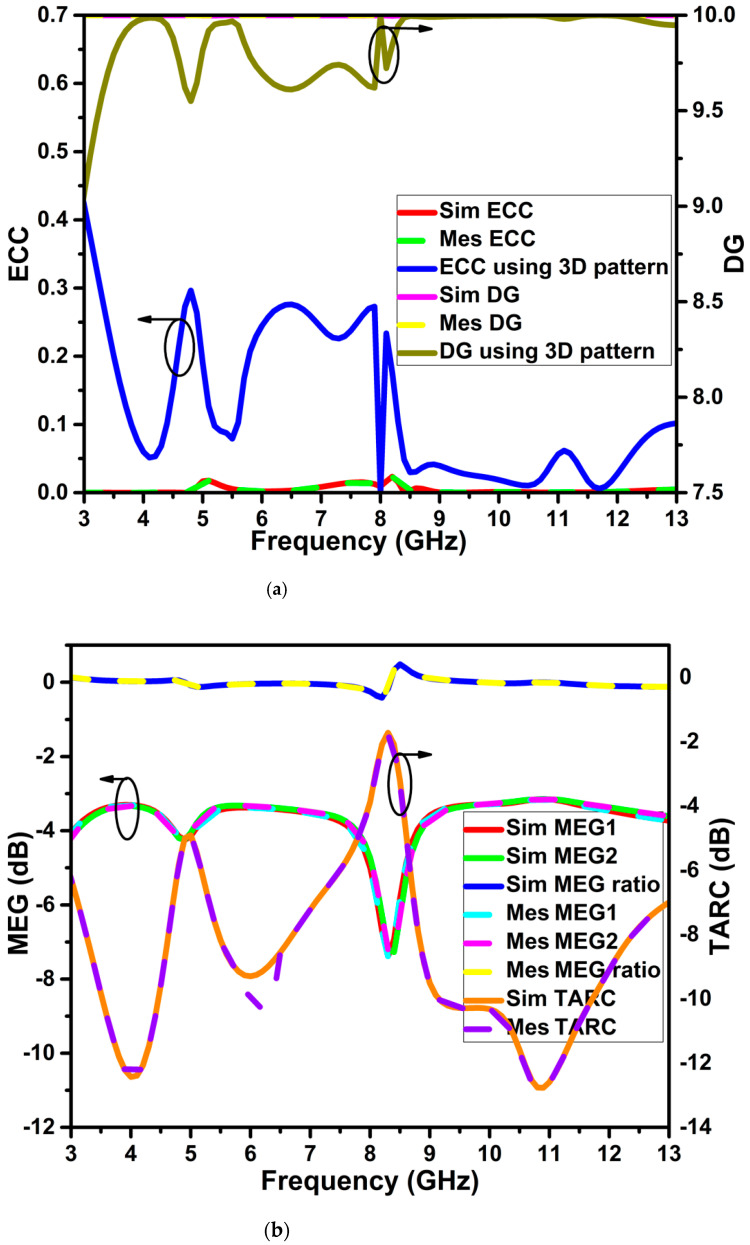

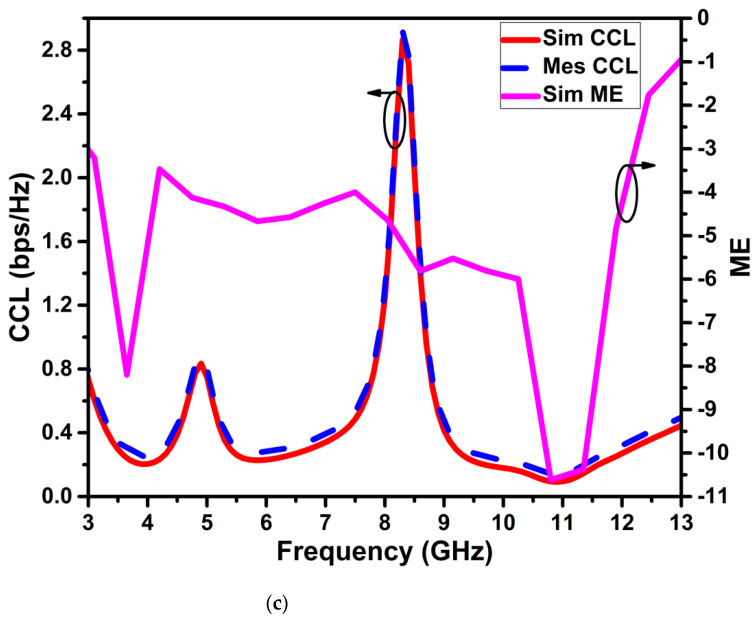
MIMO diversity metrics of the projected antenna (**a**) ECC and DG, (**b**) MEG and TARC, (**c**) CCL and ME.

The diversity properties of the proposed dual band-notch MIMO antenna are evaluated. The envelope correlation coefficient (ECC) [27] illustrates the degree of correlation or isolation among the MIMO elements. The intensity of the received pulse’s signal-to-noise ratio defines the diversity gain (DG). The accepted ideal values for the ECC and DG are less than 0.5 and close to 10 dB. The antenna’s S11 does not appropriately account for radiation efficiency and bandwidth. As a result, the total active reflection coefficient (TARC) for S11 can be employed [28]. TARC takes into account the coupling impact of the MIMO system and random signal combinations. The mean effective gain (MEG) measures the MIMO antenna’s capacity to accept electromagnetic energy in fading environments [29]. The mean received power of a test antenna in relation to the ideal antenna is known as MEG. The channel capacity grows proportionally as the number of antenna components rises. However, placing many antennas close together creates a correlation and leads to channel capacity (CCL). This figure of merit quantifies the maximum rate of the channel’s data transfer for reliable communication. The multiplexing efficiency statistic is a crucial diversity metric (ME). By calculating the efficiency (η) and degree of correlation ($\rho_{ij}$) among the system’s components, the ME describes the MIMO system. The aforementioned diversity metrics are computed using Equations (15)–(21). The MIMO diversity features of the projected antenna are represented in Figure 22.

(15) $$ECC(\rho_{ij})=\frac{{\left|\int_{0}^{2\pi }\int_{0}^{\pi }\left[XPR\cdot E_{\theta i}E_{\theta j}^{*}P_{\theta }+E_{\phi i}E_{\phi j}^{*}P_{\phi }\right]\right|}^{2}}{\int_{0}^{2\pi }\int_{0}^{\pi }\left[XPR\cdot E_{\theta i}E_{\theta i}^{*}P_{\theta }+E_{\phi i}E_{\phi i}^{*}P_{\phi }\right]d\Omega \times \int_{0}^{2\pi }\int_{0}^{\pi }\left[XPR\cdot E_{\theta j}E_{\theta j}^{*}P_{\theta }+E_{\phi j}E_{\phi j}^{*}P_{\phi }\right]d\Omega \;}\;$$

where *XPR* is the cross-polarization ratio, $P_{\theta }$ and $P_{\phi }$ are the incoming wave’s angular density functions at *θ* and *ϕ* fields, $E_{\theta }$, $E_{\phi }$ are the complex envelopes across *θ* and *ϕ* components.

(16) $$\;ECC(\rho_{ij})=\frac{-\sum_{n=1}^{N}S_{ni}^{*}\;S_{nj}}{\sqrt{\left(1-\sum_{n=1}^{N}{\left|S_{ni}\right|}^{2}\right)\left(1-\sum_{n=1}^{N}{\left|S_{nj}\right|}^{2}\right)}}\;$$

where $S_{ni}$ and $S_{nj}$ are the S-parameters of *i* and *j* ports, respectively, $S_{ni}^{*}$ is conjugate of $S_{ni}$.

(17) $$DG=10\sqrt{1-{\left|\rho_{ij}\right|}^{2}}\;$$

(18) $$\;\mathrm{TARC}(\Gamma_{a}^{t})=\frac{\sqrt{\sum_{j=1}^{4}{\left|S_{j1}+\sum_{m=2}^{4}S_{jm}e^{j\theta_{m-1}}\right|}^{2}}}{\sqrt{4}}\;$$

(19) $$\;MEG_{i}=0.5\;\eta_{i,rad}=0.5\left(1-\sum_{j=1}^{P}\left|S_{ij}\right|\right)\;$$

(20) $$CCL=-log_{2}\det{\left(\alpha \right)}^{R}$$

where $\alpha^{R}$ is the receiving antenna correlation matrix.

(21) $$\;ME(\eta_{mux})=\sqrt{\eta_{1}\eta_{2}\left(1-\rho_{ij}\right)}\;$$

Figure 22 shows the values of the diversity metrics as ECC < 0.001 (using S-parameter) and less than 0.3 (using a 3D pattern), DG close to 10 dB (using S-parameter) and >9.6 dB (using a 3D pattern), MEG < −3 dB, TARC < −10 dB, CCL < 0.3 bps/Hz, and ME <−2 dB across the operational frequency except for the notched bands.

### 5.6. Comparative Analysis

As shown in Table 7, the performance of the proposed antenna design is contrasted with past research that is currently available in the literature. The proposed design improves compactness, MIMO diversity parameters, and has relatively good time-domain properties.

## 6. Conclusions

This study presents a two-port, dual band-notch UWB antenna based on neutralization line (NL). The NL serves as a decoupling device in the connection between the two similarly situated dual band-notch UWB antennas. The NL improves the MIMO system’s isolation between the antenna elements by producing the opposing current to the coupling current. The proposed design has a simple and effective structure with compact dimensions of 0.24 × 0.31 × 0.01 λ^3^, operating in the frequency range of 3.4 to 12.1 GHz, except for the frequency band notching of 4.5–5.3 GHz and 7.2–9 GHz. The port-to-port isolation is greater than 16 dB across the impedance bandwidth. The time-domain characteristics GD is less than 1 ns except for the notched frequency bands, and FF is greater than 0.96 in side-to-side and face-to-face orientation. The MIMO diversity parameters have ECC< 0.3, DG > 9.6 dB, MEG < −3 dB, TARC < −10 dB, CCL < 0.3 bps/Hz, and ME < −2 dB across the operational frequency, all other than the serrated bands, and characteristic mode analysis are also considered for the antenna design. The projected antenna is suitable for MIMO wireless communication, according to the numerical and experimental results. Future studies will concentrate on expanding the number of sending and receiving antennas to increase the channel capacity. The proposed decoupling approach can also be integrated with other ways as a hybrid decoupling structure to improve isolation among the antenna elements.

## Figures and Tables

**Table 1:** Optimal values of the UWB antenna dimensions (mm).

Ls	Ws	b1	b2	b3	b4	b5	b6	b7	b8
21.5	13	5.6	3.8	7	7.1	2	4	3	2

**Table 2:** Optimal values of the notch elements used in band-notched UWB antenna dimensions (mm).

n1	n2	n3	n4	n5	n6
6	6	5	1.4	0.25	0.8

**Table 3:** Geometrical information of the two-port band-notch antenna (mm).

Ls	Ws	c1	c2	c3	c4	c5	c6	c7
21.5	28	16	0.5	3.8	0.5	3	6	0.8

**Table 4:** Significance of eigenvalues.

$\mathrm{Eigenvalues}\;(\lambda_{i})$	$\mathrm{CA}\;(\alpha_{i})$	Significance
$\lambda_{i\;}<0$	$180<\alpha_{i\;}<270$	Electric energy is stored in capacitors.
$\lambda_{i\;}=0$	$\alpha_{i\;}=180$	Resonating point.
$\lambda_{i\;}>0$	$90<\alpha_{i\;}<180$	Magnetic energy is stored in inductors.

**Table 5:** CMA of the projected antenna design.

Resonant Frequency	Modes	Significance	Eigenvalue	CA (Degrees)	Nature of the Mode
3.7 GHz	Mode1	0.7	1.09	132	Inductive
	Mode2	0.08	−11.80	265	Capacitive (Nonsignificant)
6.8 GHz	Mode1	0.98	−0.21	192	Capacitive
	Mode2	0.89	−0.50	207	Capacitive
11.5 GHz	Mode1	0.97	−0.24	193	Capacitive
	Mode2	0.99	−0.07	183	Capacitive
	Mode3	0.80	−0.74	216	Capacitive
	Mode4	0.89	0.48	153	Inductive

**Table 6:** The FF values at different ports and orientations.

Orientation	Ports	FF
Face to Face	(1, 3)	0.98
	(1, 4)	0.97
Side to Side	(1, 3)	0.96
	(1, 4)	0.97

**Table 7:** Performance comparison of the projected antenna with the designs available in the literature.

Ref.	Techniques Used	Dimensions (λg3, at a Center Frequency)	Impedance Bandwidth (GHz)	Isolation (dB)	ECC	DG	MEG	TARC	CCL	ME
[17]	DGS	1.2 × 0.9 × 0.05	2–10.6	>22	<0.1	-	-	-	-	-
[30]	Stub and DGS	2.5 × 1.1 × 0.01	2.57–12.2	>15	<0.005	≈10	-	-	<0.4	-
[31]	DGS	1.0 × 1.2 × 0.06	3.18–11.26	>22	<0.06	<9.7	-	-	-	-
[32]	DGS with EBG	1.0 × 1.2 × 0.03	3.1–11	>25	<0.01	>9.99	-	-	<0.1	-
[33]	Neutralization line	0.7 × 0.7 × 0.01	3.1–5	>22	<0.1	-	-	-	-	-
[34]	DGS and parasitic element	1.5 × 1.2 × 0.03	3–11	>20	<0.02	≈10	-	-	-	-
[35]	parasitic element and EBG	1.6 × 1.3 × 0.06	3–10.7	>25	<0.001	≈10	-	-	-	-
proposed	Neutralization line	0.9 × 1.2 × 0.06	3.4–12.1	>16	<0.001	≈10	<−3	<−10	<0.3	<−2

- Not available.
